# Copper catalysis in organic synthesis

**DOI:** 10.3762/bjoc.11.244

**Published:** 2015-11-19

**Authors:** Sherry R Chemler

**Affiliations:** 1 Department of Chemistry, Natural Sciences Complex, SUNY Buffalo, Buffalo, NY 14260, USA

**Keywords:** copper catalysis

Copper is a very versatile transition metal that has been used as a building material by human civilizations for over 6000 years. Copper is also an essential element, responsible for important biological processes. The title of this Thematic Series published in the Beilstein Journal of Organic Chemistry is “Copper catalysis in organic synthesis”. A web of science^TM^ topic search of “copper-catalyzed synthesis” indicated over 500 papers had been published in 2014. In point of fact, there has been a steady increase in publications on this topic since 1988. In the late 1980’s and early 1990’s, the topics centered on copper nitrene reactivity (e.g., aziridination), copper carbene chemistry, conjugate additions and cross-coupling reactions. Some of the most highly cited papers of all time on this topic are reviews on conjugate addition, cross-coupling, and [3 + 2] “Click” reaction applied to bioconjugation.

The growth in copper-catalyzed organic reactions may be driven by a couple of factors. First, copper chemistry is incredibly diverse. Depending on its oxidation state, this metal can efficiently catalyze reactions involving both one and two-electron (radical and polar) mechanisms, or both. Copper coordinates easily to heteroatoms and to π-bonds and is well-known to activate terminal alkynes. The Ullman and Goldberg C–C and C–N cross-coupling reactions were discovered over a century ago and their development has really blossomed over the past twenty years. Second, copper is an earth-abundant metal, making its use more cost effective and more sustainable than precious transition metal catalysts.

Over 25 contributions from leaders in the field of copper-catalysis from 7 countries make up this Thematic Series, “Copper catalysis in organic synthesis”. Contributions include authoritative reviews on the latest developments in copper-catalyzed Click applications, C–N cross-coupling, C–H functionalization, trifluoromethylations, asymmetric Ullmann and Goldberg couplings, asymmetric acetylide additions to carbonyl groups, radical alkylations and asymmetric conjugate additions as well as original contributions in the area of copper-catalyzed C–C cross-coupling, conjugate additions, allylic alkylations, alkene difunctionalizations, heterocycle synthesis, amide bond formation and photochemical Click reaction development.

I am grateful to the contributors and the reviewers who participated in the production of this Thematic Series. It is clear from the impact copper catalysis has had on organic synthesis that copper should be considered a first line catalyst for many organic reactions. I anticipate further growth in the field in the years to come.

Sherry R. Chemler

Buffalo, November 2015

